# Special Hospitals

**Published:** 1893-08-26

**Authors:** 


### The Institutional Workshop.

### HOSPITAL ADMINISTRATION.

### II.—SPECIAL HOSPITALS.

In the previous article we brought out the percentage of the cost of administration as compared with that of maintenance at the principal general hospitals of the metropolis, having one hundred beds and upwards. We propose to-day to follow up our enquiries by dealing with the special hospitals on the same plan. It is very difficult to determine what constitutes a fair percentage of the cost of administration in the case of special hospitals. Most of these institutions are relatively small, and they have to maintain themselves by increased energy and continuous and extensive appeals. It has also to be remembered that there are special hospitals, and special hospitals; thus, hospitals for consumption, hospitals for children, hospitals for the paralysed, and certain ophthalmic and fever hospitals, as well as the Royal Sea Bathing Infirmary, Margate, and the London Temperance, are all well entitled, from the number of beds they contain, to be classed as hospitals. When we come, however, to certain other groups which contain from twenty to sixty beds, we have to deal with institutions which are relatively so small as to be at best only cottage hospitals, and in many cases to be little better, if at all, than large out-patient departments with a few beds for certain cases, which may possess special interest or urgency, and so need in-patient treatment. Of course the question of size need have little or nothing to do with the claim of such institutions upon public support, though it has much to do with the system upon which their expenditure should be dealt with. We are of opinion that no hospital containing less than one hundred beds should mix the cost of the in-patients with that of the out-patients. In these cases the out-patients absorb very often two-thirds or more of the whole expenditure, and so it is desirable that any outlay upon the out-patient department should be kept distinct in the accounts from that expended upon the in-patient branch of the work.

Last year (*vide* The Hospital for September 3rd, 1892, page 381) we made similar suggestions for a modification in the method of rendering accounts in the best interests of these relatively small hospitals, because, by its adoption, the managers would at once place themselves in the strongest possible position should any comparison ever be attempted between the cost of the work they do and that which is undertaken by institutions where the bulk of the expenditure is made upon the in-patient department. Unfortunately, so far as our observation goes, very few, if any, of the special hospitals have so far had the wisdom to make arrangements for keeping the expenditure of the in-patients wholly distinct from that of the out-patients

#### The Cost of an Out-Patient.

In the hope of securing this reform in the methods adopted by special hospitals, we have consulted those whose opinion is entitled to weight, and after due deliberation have come to the conclusion that on the whole it is reasonable and just, so far as present information goes, to estimate the cost of each out-patient at the various classes of hospitals at the following rates: General hospitals in London, Provinces, Scotland, and Ireland, 1s. 6d. per out-patient; consumption, cancer, and ear and throat hospitals, 3s.; children's and ophthalmic hospitals, 1s. 6d.; women's hospitals, 2s.; hospitals for paralysis, epilepsy, &c., 5s.; lying-in hospitals, 10s. It may further be borne in mind that certain diseases entail a specially heavy outlay, and, with a view of arriving at a fair basis of comparison, it is necessary to classify the special hospitals into groups, under their special headings, so that all those which deal with the same class of disease may easily be compared with each other.

Group I.—Metropolitan Consumption Hospitals.

| Name of Hospital. | Beds for Occupation. | Average Number Occupied. | Total In-patients Admitted. | Total Out-patients Admitted. | Percentage of cost of Administration to that of Maintenance. | Percentage of cost of Administration to Total Expenditure. |
|---|---|---|---|---|---|---|
| Brompton for Consumption | 321 | 270 | 1,602 | 15,357 | 7·646 | 7·099 |
| City of London for Diseases of the Chest, Victoria Park | 164 | 112 | 1,079 | 14,865 | 10·419 | 9·436 |
| Royal National Ventnor ... | 134 | 109 | 747 | None. | 8·914 | 8·184 |
| Royal for Diseases of the Chest, City Road... ... | 80 | 45 | 542 | 6,231 | 28·835 | 22·381 |
| North London, Hampstead | 50 | 45 | 371 | 2,899 | 30·170 | 23·163 |

The figures in the foregoing table reveal at once the fact that in the case of a special hospital, intended for the treatment of consumption, it is desirable, for economical reasons, to provide at least one hundred beds. This is apparent from the fact that whereas the three larger hospitals in the table expend less together than 10 per cent. upon administration as compared with maintenance, the two smaller institutions, which contain eighty and fifty beds respectively, of which forty four and forty-seven beds respectively were occupied throughout the year, cost in each case for administration more than three times as much as the Hospital for Consumption, Brompton. We commend these figures to the consideration of the committees and the public. We would remind both that in the case of the North London, Hampstead, at any rate, there is a splendid site not yet occupied by buildings, and that London at the present time urgently needs many hundreds more hospital beds for the reception of cases of phthisis. There are in the metropolis, and throughout the country—and the question of providing an adequate number of beds for consumptive cases is a country question quite as much, if not more, than it is a metropolitan one, because so many of the patients in the consumption hospitals are at present sent from the country—an increasing number of wealthy people who desire to give of their abundance substantial sums for the alleviation of suffering in their day and generation. We know no better object upon which they can expend a very large sum of money than by placing themselves in communication with the Chairman of the North London Consumption Hospital, Hampstead, with the object of concerting measures to considerably extend the number of beds at present available in these institutions for the reception and treatment of urgent cases of consumption.

### Cost of Management Compared.

The addition of the percentage of the cost of administration to total expenditure again shows that there is little point in the contention of those who maintain it to be unfair to make a comparison between the various hospitals on the basis of the percentage of the cost of administration to that of maintenance. It is satisfactory to note that the Brompton Consumption and the Royal National Hospital, Ventnor, both show a considerable decrease in the percentage of cost during 1892, as compared with 1891, whilst the expenditure at the City of London Hospital for Diseases of the Chest shows little alteration. At the Royal Hospital for Diseases of the Chest and the North London Hospital for Consumption the cost of administration shows a tendency to steadily, and in the first instance, to largely increase. Seeing that nearly 5s. 6d. out of every sovereign at the Royal Chest Hospital, and upwards of 6s. out of every sovereign at the North London Hospital, is sunk in administering to the expenses, we are strongly of opinion that the committees of these institutions should look to their guns, and that, in conjunction with their officers, they should take steps, as the Hampstead Hospital Committee have evidently done, which will lead to a speedy and material reduction in the charges. It is true, no doubt, that these two consumption hospitals contain relatively few beds, but when all due allowance has been made for this circumstance, the fact remains that the outlay upon administration at both institutions is quite 10 per cent. more than it should be. We feel it our duty to direct special attention to this point, and we shall hope in due course to receive some explanation of the causes which have produced results so unfavourable to the management when compared with that of the larger hospitals included in the table which belong to the same group.

